# ID3 mediates BMP2-induced downregulation of ICAM1 expression in human endometiral stromal cells and decidual cells

**DOI:** 10.3389/fcell.2023.1090593

**Published:** 2023-02-24

**Authors:** Jin Luo, Yaqin Wang, Hsun-Ming Chang, Hua Zhu, Jing Yang, Peter C. K. Leung

**Affiliations:** ^1^ Reproductive Medicine Center, Hubei Clinic Research Center for Assisted Reproductive Technology and Embryonic Development, Renmin Hospital of Wuhan University, Wuhan, China; ^2^ Department of Obstetrics and Gynaecology, BC Children’s Hospital Research Institute, University of British Columbia, Vancouver, BC, Canada; ^3^ Reproductive Medicine Center, Department of Obstetrics and Gynecology, China Medical University Hospital, Taichung, Taiwan

**Keywords:** recurrent pregnancy loss (RPL), endometrium, BMP2, ICAM1, Id3

## Abstract

Recurrent pregnancy loss (RPL) remains an unsolved problem in obstetrics and gynecology, and up to 50% of RPL cases are unexplained. Unexplained RPL (uRPL) is widely considered to be related to an aberrant endometrial microenvironment. BMP2 is an important factor involved in endometrial decidualization and embryo implantation, and intercellular adhesion molecule 1 (ICAM1) is a critical inflammatory regulator in the endometrium. In this study, we found that endometrial samples obtained from Unexplained RPL patients have significantly lower BMP2 and higher ICAM1 levels than fertile controls. For further research on the relationship between BMP2 and ICAM1 and the potential molecular mechanisms in Unexplained RPL, immortalized human endometrial stromal cells (HESCs) and primary human decidual stromal cells (HDSCs) were used as study models. Our results showed that BMP2 significantly decreased ICAM1 expression by upregulating DNA-binding protein inhibitor 3 (ID3) in both HESCs and HDSCs. Using kinase receptor inhibitors (dorsomorphin homolog 1 (DMH-1) and dorsomorphin) and siRNA transfection, it has been found that the upregulation of ID3 and the following downregulation of ICAM1 induced by BMP2 is regulated through the ALK3-SMAD4 signaling pathway. This research gives a hint of a novel mechanism by which BMP2 regulates ICAM1 in the human endometrium, which provides insights into potential therapeutics for unexplained RPL.

## Introduction

Recurrent pregnancy loss (RPL), which is defined as the loss of two or more pregnancies before 24 weeks of gestation in the same couple based on the European Society of Human Reproduction and Embryology (ESHRE) guideline (ESHRE Guideline Group on RPL et al., 2018), affects approximately 5% of couples trying to conceive (Garrido-Gimenez and Alijotas-Reig, 2015). Although various etiological factors have been shown to lead to RPL (El Hachem et al., 2017), nearly 50% of RPL cases cannot find identifiable causes or risk factors, which are classified as unexplained RPL (uRPL) (Garrido-Gimenez and Alijotas-Reig, 2015; El Hachem et al., 2017). The profound negative impact of RPL on both physical and mental health combined with the uncertainties in regard to the etiology and management options makes uRPL an ongoing challenge for both the clinical and scientific community. Therefore, exploring the pathogenesis of uRPL is essential for developing early interventions and ensuring a better pregnancy outcome.

As a unique tissue that indirectly contacts with the embryo, the endometrium plays a vital role in the establishment and maintenance of pregnancy. In recent decades, extensive research has been carried out to elucidate the biomolecular mechanisms that promote the acceptance of the embryo by the endometrium and the specific molecules and cellular pathways involved in endometrial receptivity. A number of studies have shown that various factors are dysregulated in the endometrium of women with uRPL (Comba et al., 2015; Dambaeva et al., 2021; Heidari et al., 2021; Zhu et al., 2022), including intercellular adhesion molecule 1 (ICAM1). ICAM1 is a single-chain, 90 kDa inducible cell-surface glycoprotein and a member of the immunoglobulin superfamily (Rothlein et al., 1991). In the human endometrium, ICAM1 is localized to the apical surface of the glandular epithelium, the vascular endothelium, and endometrial stromal cells throughout the menstrual cycle, and its expression in stromal cells is upregulated during menstruation (Thomson et al., 1999). The expression of ICAM1 has also been found in first-trimester human decidual stromal cells (Marzusch et al., 1993). Studies have found that inappropriate expression of ICAM1 may contribute to various gynecological and obstetric disorders, including endometriosis (Pino et al., 2009), gestational diabetes mellitus (Xie et al., 2008), preeclampsia (Austgulen et al., 1997) and RPL (Comba et al., 2015). The wide distribution of ICAM1 and these findings indicate that ICAM1 is involved in the menstrual process, glands, blood vessels and stroma function in the human endometrium and plays a crucial role in a successful pregnancy. However, the regulatory mechanism of ICAM1 during the menstrual cycle and early pregnancy in the human endometrium and decidua is largely unknown.

Bone formation protein 2 (BMP2) is a member of the transforming growth factor β (TGF-β) superfamily which serves as a key regulator of both endometrial degeneration (Li et al., 2007; Stoikos et al., 2008) and trophoblast cell invasion (Zhao et al., 2018a; Zhao et al., 2018b; Zhao et al., 2020). Functionally, Sma- and Mad-related (SMAD) proteins SMAD1/5/8 are phosphorylated when BMP2 binds to the TGF-β type II receptor and recruits the TGF-β type I receptors (ALK2, ALK3 and ALK6). After associating with the SMAD4 protein, the activated SMAD1/5/8 complex migrates to the nucleus wherein it regulates the target genes expression (Zhao et al., 2018a; Zhang et al., 2020; Luo et al., 2021a; Luo et al., 2021b). BMP2 is significantly elevated during decidualization in immortalized human endometrial stromal cells (HESCs) and primary human endometrial stromal cells (HDSCs) in response to steroid hormones and cyclic adenosine monophosphate (cAMP) (Luo et al., 2020). In addition, exogenous BMP2 treatment promotes the decidual response of these two kinds of cells (Luo et al., 2020). Our previous studies also demonstrated that BMP2 plays an essential role in endometrial stromal remodeling (Luo et al., 2020). However, the expression of BMP2 in the endometrium of patients with RPL has not been reported. We hypothesize that the overexpression of ICAM1 in the endometrium of uRPL patients may be controlled by BMP2 given the spatiotemporal variations in the expression of BMP2 and ICAM1 in the human endometrium throughout the menstrual cycle and pregnancy. To test this hypothesis, we analyzed the endometrial BMP2 and ICAM1 expression levels between uRPL patients and healthy women and explored the underlying molecular mechanisms and signaling pathways using HESCs and primary HDSCs.

## Materials and methods

### Patient recruitment and tissue collection

The use of endometrial tissue in the research received clearance from the ethics committee of Renmin Hospital, Wuhan University. A total of 16 women diagnosed with uRPL and 12 normal fertile women were recruited. The inclusion and exclusion criteria refer to previously published literature (Comba et al., 2015; Benner et al., 2022). Briefly, uRPL was defined as two or more fetal losses before 24 weeks of gestation without known causes of miscarriages. The control group was made up of normal fertile women with regular periods who had had at least one live birth and no spontaneous miscarriages in the past. The exclusion criteria were the use of immunosuppressive drugs, steroid hormones, antibiotics, diabetes mellitus and smoking. Endometrial biopsies were obtained from women who attended the reproductive center of Renmin Hospital of Wuhan University and received a endometrial biopsy on day 21 or day 22 of menstrual cycle which were identified as mid-secretory phase by pathological examination. In order to isolate primary HDSCs, first-trimester decidual specimens (between the 7th and 12th weeks of gestation) were collected from healthy women who were having an elective abortion as part of the CARE Program at the BC Women’s Hospital and Health Centre. The research was authorized by the University of British Columbia’s Research Ethics Board. All participants in this study were between 20 and 40 years of age and provided written informed consent.

### Cell models

Considering that both BMP2 and ICAM1 are expressed in human endometrial stromal cells throughout the menstrual cycle, and endometrial decidualization is a critical physiological event in the female menstrual cycle, thus immortalized human endometrial stromal cells (HESCs; ATCC^®^ CRL-4003) and primary HDSCs were used as study cell models, representing non-decidual and decidual stromal cells respectively, to systematically study the regulatory effect of BMP2 on ICAM1 in human endometrium at different stages of the menstrual cycle.

### Culture and treatment of immortalized HESCs

HESCs were grown in DMEM/F12 medium without phenol red (Sigma-Aldrich, St. Louis, MO, United States of America), added with 10% charcoal dextran-treated fetal bovine serum (FBS; HyClone Laboratories, Inc., Logan, UT, United States of America), 1% ITS - Premix (BD Biosciences, San Jose, CA, United States of America) and 5 ng/mL puromycin (Thermo Fisher Scientific, Ottawa, ON, CAN). Every culture was kept at 37 °C in an incubator with 5% CO_2_. HESCs were cultivated for a day after being seeded at a density of 4 × 10^5^ cells per plate in 60-mm tissue culture dishes with full culture media. HESCs were treated with BMP2 (0, 10, 25, or 50 ng/ml) for the concentration-dependent research or with 25 ng/mL BMP2 for the time-course study after serum deprivation in DMEM/F12 media without FBS for 18 h. For the concentration-dependent investigation, cells were taken at 24 h, while for the time-course study, cells were taken at 3, 6, 12, 24 h, and 48 h.

### Isolation and cultivation of primary HDSCs

Primary HDSCs were separated from decidual tissues by means of enzymatic dispersion and mechanical dissociation, as mentioned before (Zhu et al., 2007). Generally, the samples were washed in cold phosphate-buffered saline (Gibco, Life Technologies, Inc., Carlsbad, CA, United States of America) three times, and then minced and treated with 0.1% collagenase (type IV; Sigma‒Aldrich), 0.1% hyaluronidase (type I-S; Sigma‒Aldrich) and 0.5 mg/ml DNase I (Sigma‒Aldrich) and subsequently digested in a shaking water bath for 60 min at 37°C. The supernatant was neutralized by the addition of phenol red-free DMEM/F12 medium supplemented with 10% FBS before the cells were passed through a 40 m nylon filter (BD Biosciences, Bedford, UK). The undigested tissue fragments were left on the filter, and the stromal cell-containing eluate was transferred into a 50 ml tube. The cells were then pelleted by centrifuging them at 1200 g for 3 min at room temperature. Following that, the cell pellets were washed, resuspended, and seeded in phenol red-free DMEM/F12 media with antibiotics (100 U/ml penicillin and 100 μg/ml streptomycin, Life Technologies, Inc.), 10% FBS, 30 nM 17β estradiol (E2; Sigma Aldrich), and 1 μM progesterone (P4; Sigma Aldrich). All decidual stromal cell cultures were afterwards maintained at 37°C in a humid incubator with 5% CO2, unless otherwise stated, in this culture medium. The purity of the HDSCs was determined by immunofluorescent staining for vimentin and cytokeratin-7 as described previously (Zhu et al., 2007). HDSCs were cultivated at a density of 5 × 10^5^ cells per plate in 60-mm tissue culture dishes for the time- and concentration-dependent studies, and BMP2 was added in the same manner as HESCs.

### Antibodies and reagents

Recombinant human BMP2, dorsomorphin homolog 1 (DMH-1) and dorsomorphin dihydrochloride (DM) were obtained from R&D Systems (Minneapolis, MN, United States of America). Monoclonal rabbit anti-DNA-binding protein inhibitor 3 (ID3), monoclonal mouse anti-ICAM1 and polyclonal rabbit anti-SMAD4 antibodies were obtained from Cell Signaling Technology (Beverly, MA, United States of America). Santa Cruz Biotechnology supplied the monoclonal mouse GAPDH antibody sc-47,724 (Santa Cruz, CA, United States of America). Bio-Rad Laboratories, Inc. supplied goat anti-mouse and goat anti-rabbit IgG that had been conjugated with horseradish peroxidase (Hercules, CA, United States of America).

### Reverse transcription-quantitative real-time PCR (RT‒qPCR)

Total RNA was extracted from collected endometrial tissue or cultured cells using TRIzol reagent (Invitrogen, Life Technologies, Inc.) according to the manufacturer’s instructions. Each reverse transcription procedure used 2 μg of RNA to create first-strand complementary DNA (cDNA) utilizing random primers and Moloney murine leukemia virus reverse transcriptase (Promega, Madison, WI, United States of America). Using an Applied Biosystems 7300 Real-Time PCR System, SYBR Green or TaqMan was used for RT-qPCR assays. Each 25 μl qPCR reaction comprised 12.5 μl of SYBR Green PCR Master Mix (Applied Biosystems, Foster City, CA), 100 ng of cDNA, and 7.5 nM of each specific primer. These primers were used in this study: ICAM-1, 5′-CTC​CAA​TGT​GCC​AGG​CTT​G-3' (forward) and 5′- 5′-CAG​TGG​GAA​AGT​GCC​ATC​CT-3’ (reverse); ID3, 5′- CAG​CTT​AGC​CAG​GTG​GAA​ATC​C-3' (forward) and 5′-GTC​GTT​GGA​GAT​GAC​AAG​TTC​CG-3’ (reverse); SMAD4, 5′-TGGCCCAGGATCAGT AGGT-3′ (forward) and 5′-CAT​CAA​CAC​CAA​TTC​CAG​CA-3′ (reverse); and GAPDH, 5' -GAG​TCA​ACG​GAT​TTG​GTC​GT-3' (forward) and 5'- GAC​AAG​CTT​CCC​GTT​CTC​AG-3' (reverse). Alternatively, TaqMan gene expression assay kits for BMP2 (Hs00154192_m1), ALK2 (Hs00153836_m1), ALK3 (Hs01034913_g1), and GAPDH (Hs02758991_g1) were bought from Applied Biosystems. Each 20 μL TaqMan RT**-**qPCR reaction comprised 1 × TaqMan Gene Expression Master Mix (Applied Biosystems), 20 ng of cDNA, and 1 × specific TaqMan assay Mix containing primers and probes. Relative quantification of the mRNA levels of target genes was determined based on the comparative cycle threshold (Ct) method, and the 2^−ΔΔCt^ method was used specifically, with the results standardized to endogenous GAPDH.

### Western blot analysis

Total protein extracts were produced using lysis buffer (Cell Signaling Technology) containing protease inhibitor cocktail (Sigma Aldrich) from homogenized endometrial tissues or cultured cells. A DC Protein Assay (Bio-Rad Laboratories, Inc.) was used to measure the protein concentrations. Proteins of same concentration were put onto gels and transferred onto polyvinylidene fluoride (PVDF) membranes (Bio-Rad) after being separated by sodium dodecyl sulfate‒polyacrylamide gel electrophoresis. After blocking the membranes for 1 h at room temperature in Tris-buffered saline containing 0.1% Tween-20 (TBST) and 5% nonfat milk, they were immunoblotted overnight at 4°C with the appropriate primary antibodies. After three washes with TBST, the membranes were incubated with peroxidase-conjugated secondary antibodies for 1 hour. Enhanced chemiluminescent or SuperSignal West Femto chemiluminescent substrates (Thermo Fisher Scientific) were used to identify immunoreactive bands, which were subsequently subjected to X-ray film (Thermo Fisher Scientific). Image-Pro Plus software was used to calculate the band intensities (v4.5; Media Cybernetics, Carlsbad, CA).

### Small interfering RNA transfection

RNA interference was enabled by the transfection of small interfering RNA (siRNA). ON-TARGET plus non-targeting control pool siRNA or an ON-TARGET plus SMART pool targeting ALK2, ALK3, ID3 and SMAD4 were purchased from Dharmacon Inc. A total of 2 × 10^5^ HESCs or primary HDSCs were simultaneously seeded with full culture media 1 day before transfection. Lipofectamine RNA iMAX (Life Technologies) was used to transfect control siRNA or siRNA against ALK2, ALK3, ID3 and SMAD4 into the cells at a dose of 25 nM in accordance with the manufacturer’s instructions. The cells were then cultured for 24 h at 37 °C in a CO2 incubator untill starvation (synchronization of all the cells to the same cell cycle phase and removal of various ligands in serum). RT‒qPCR or Western blot analysis was used to assess the knockdown effectiveness of each target.

### Statistical analysis

All statistical analyses were conducted using PRISM software (GraphPad Software, Inc., San Diego, CA). Using the unpaired Student’s t-test, comparisons were made between two sets of independent samples. Multiple comparisons of means were examined using one-way ANOVA and Newman‒Keuls testing. The results are reported as the mean ± S.E.M. of at least three independent experiments. The significance threshold was established at *p* < 0.05.

## Results

### Decreased BMP2 and increased ICAM1 expression in the endometrium of patients with uRPL

**FIGURE 1:**
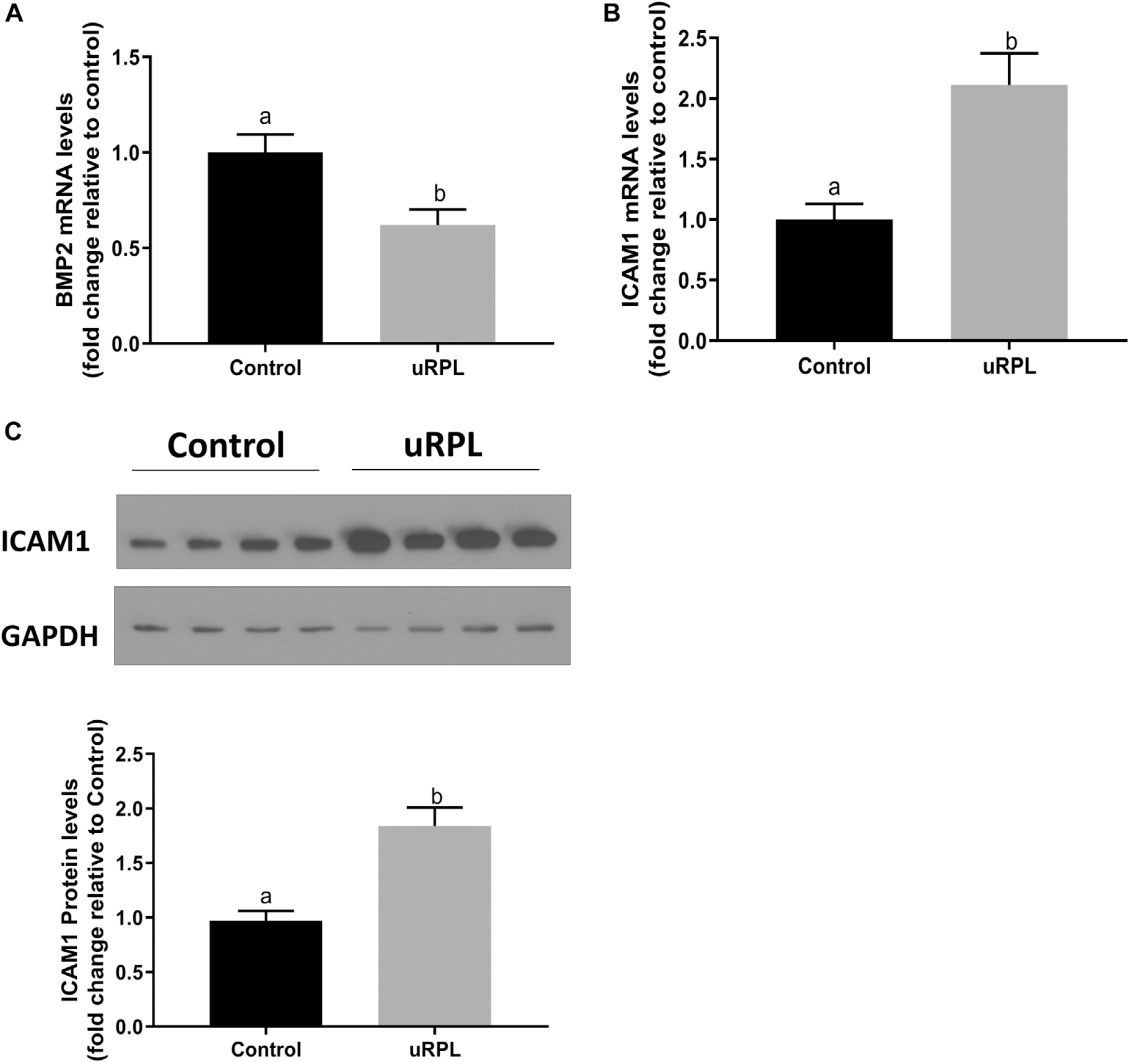
Expression levels of BMP2 and ICAM1 in the endometrium obtained from patients with unexplained recurrent pregnancy loss (uRPL) and fertile women (as a control). The endometrial samples were obtained from 12 uRPL patients and 16 fertile women (Control) during their mid-secretory phases. **(A,B)** The mRNA levels of BMP2 **(A)** and ICAM1 **(B)** of the endometrial tissues obtained from uRPL and controls were examined using RT-qPCR. **(C)** The protein levels of ICAM1 of the endometrial tissues obtained from uRPL and controls were examined using Western blot analysis. The results are expressed as the mean ± S.E.M. Different letters indicate a significant difference (*p* < 0.05).

High expression levels of BMP2 and ICAM1 have been reported in human endometrium in prior studies (Thomson et al., 1999; Bai et al., 2020). Here, the expression levels of these two factors in secretory endometrial tissues were compared between uRPL patients and healthy fertile women by using RT‒qPCR and Western immunoblotting. As shown in Figure 1, compared with normal fertile controls, the expression of BMP2 in the endometrial tissues of uRPL patients was dramatically lower at mRNA level (Figure 1A), whereas the expression of ICAM1 at both mRNA (Figure 1B) and protein (Figure 1C) levels were considerably increased in the endometrium of women with uRPL.

### BMP2 suppresses the expression of ICAM1 in HESCs and primary HDSCs

**FIGURE 2:**
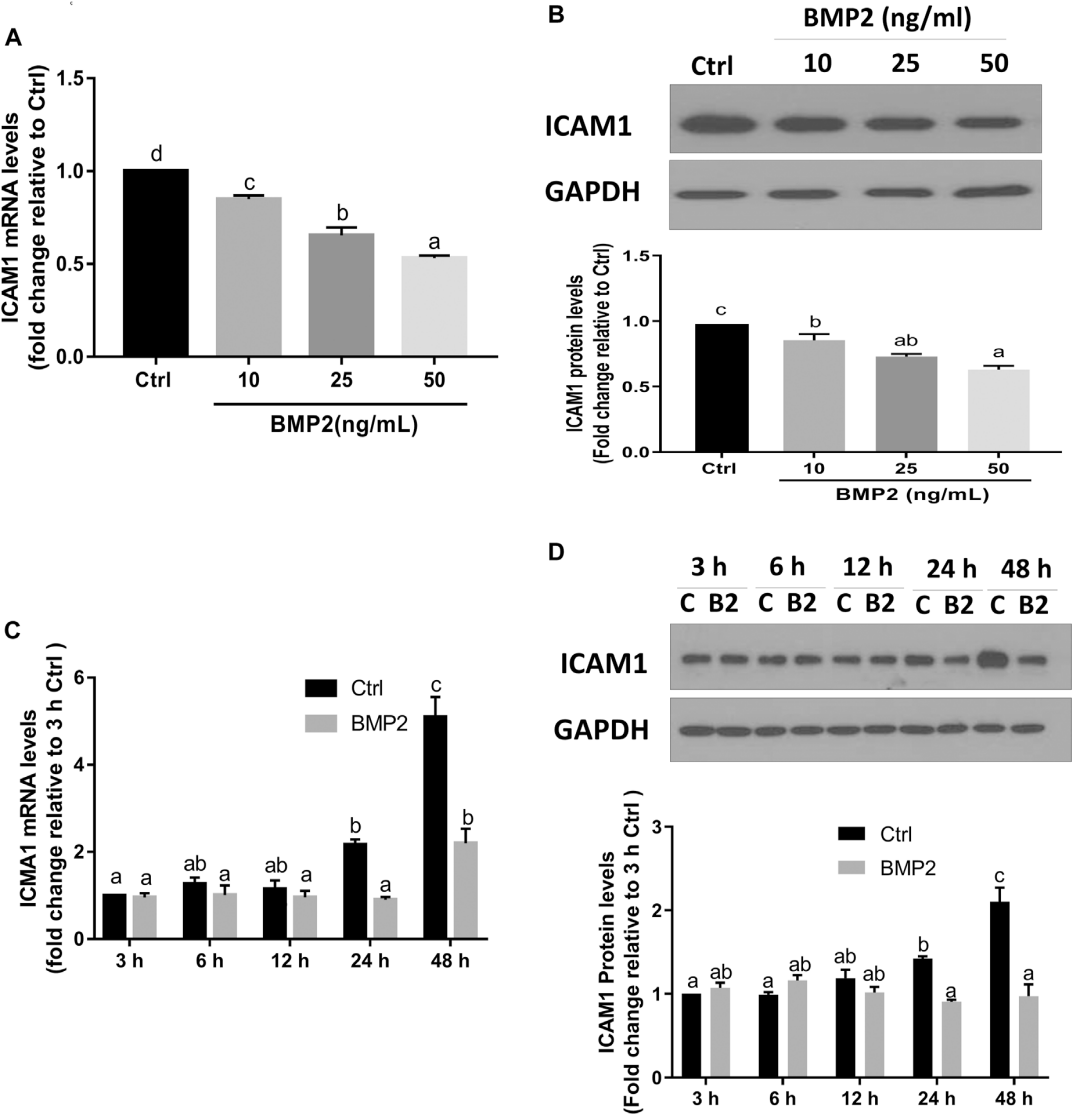
BMP2 downregulates the expression of ICAM1 in non-decidualized HESCs. **(A,B)** HESCs were treated with different concentrations (0, 10, 25, 50 ng/mL) of recombinant human BMP2 (BMP2) for 24 h, and the mRNA **(A)** and protein **(B)** levels of ICAM1 were examined using RT-qPCR and Western blot analysis, respectively. **(C,D)** HESCs were treated with 25 ng/mL of BMP2 for 3, 6, 12, 24 or 48 h, and the mRNA **(C)** and protein **(D)** levels of ICAM1 were examined using RT-qPCR and Western blot analysis, respectively. The results are expressed as the mean ± S.E.M. of at least three independent experiments. Different letters indicate significant a difference (*p* < 0.05). In detail, if the letters on two columns are different (E.g., “a” vs. “b” or “b” vs. “c”), it means that the difference between the two groups is significant, on the other hand, if the letters on the column of two groups are the same (E.g., “a” vs. “a” or “b” vs. “b”), it means there is no significant difference between two groups. C, Ctrl, Control; B2, BMP2.

**FIGURE 3:**
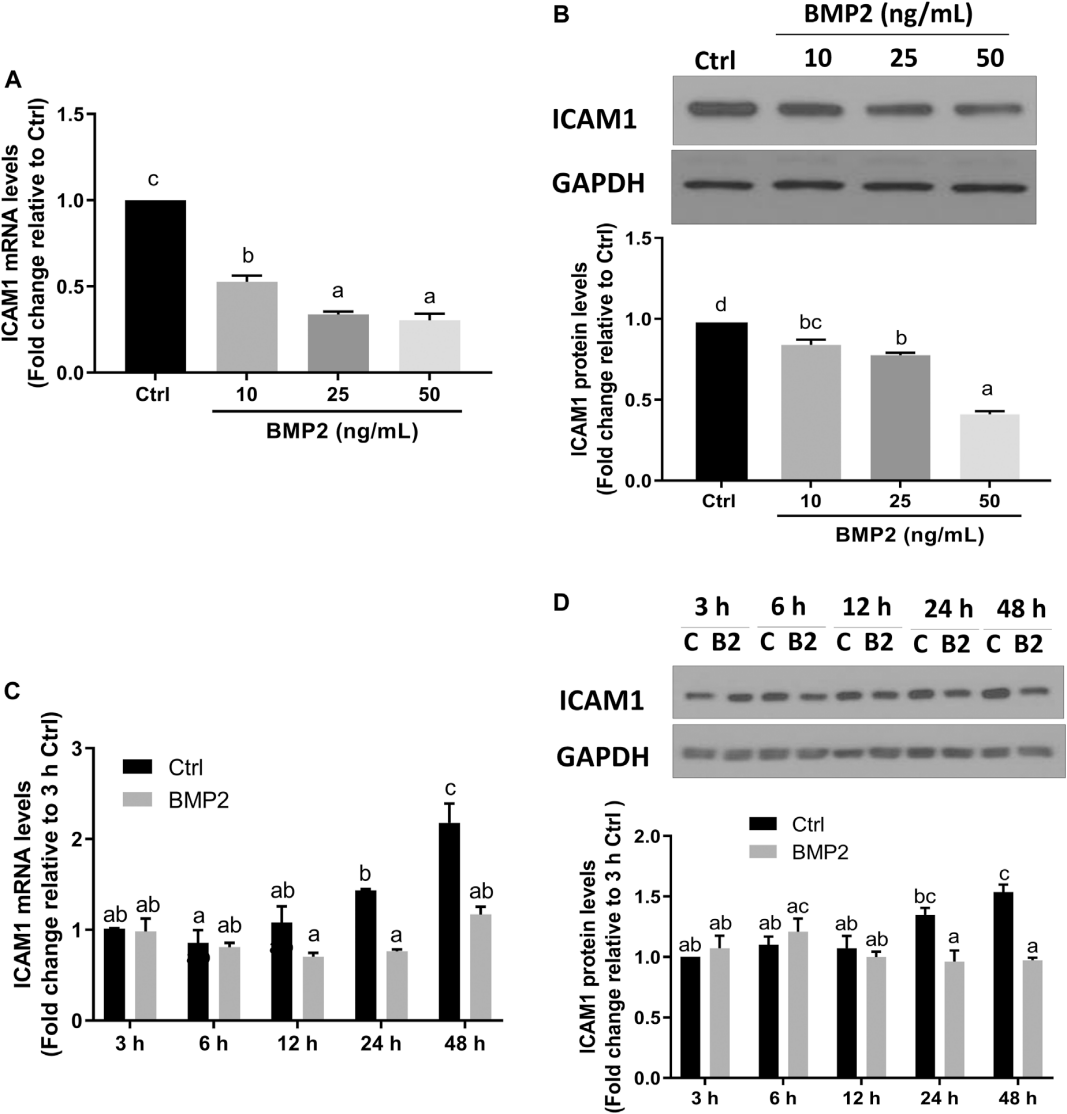
BMP2 downregulates the expression of ICAM1 in primary human endometrial stromal cells (HDSCs). (A,B) HDSCs (n = 3) were treated with different concentrations (0, 10, 25, 50 ng/mL) of BMP2 for 24 h, and the mRNA **(A)** and protein **(B)** levels of ICAM1 were examined using RT-qPCR and Western blot analysis, respectively. **(C,D)** HDSCs (n = 3) were treated with 25 ng/mL of BMP2 for 3, 6, 12, 24 or 48 h, and the mRNA **(C)** and protein **(D)** levels of ICAM1 were examined using RT-qPCR and Western blot analysis, respectively. The results are expressed as the mean ± S.E.M. Each experiment was performed in duplicate. Different letters indicate a significant difference (*p* < 0.05). C, Ctrl, Control; B2, BMP2.

To investigate if BMP2 affects ICAM1 expression in HESCs, serum-starved cells were initially treated with vehicle control or various concentrations (10, 25, or 50 ng/ml) of recombinant human BMP2 for 24 h. The expression levels of ICAM1 were detected by RT‒qPCR and Western immunoblotting. The results revealed that both the mRNA (Figure 2A) and protein levels (Figure 2B) of ICAM1 were markedly downregulated in a concentration-dependent fashion in response to the BMP2 treatment in HESCs. The time-course study showed that cultivation with 25 ng/mL BMP2 dramatically decreased the mRNA (Figure 2C) and protein levels of ICAM1 at 24 h and 48 h (Figure 2D). We also evaluated the influence of BMP2 on the expression of ICAM1 in primary HDSCs in addition to HESCs. The purity of HDSCs was determined by immunofluorescent staining with markers specific to mesenchymal cells (vimentin) and epithelial cells (cytokeratin-7). HDSCs used in these studies were approximately 98% pure as assessed by vimentin-positive and cytokeratin-negative staining (Supplementary Figure S1). In accordance with the findings in HESCs, treatment with various dosages (10, 25, or 50 ng/mL) of BMP2 for 24 h dramatically decreased the mRNA (Figure 3A) and protein (Figure 3B) levels of ICAM1 in a concentration-dependent way. The time-course analysis revealed that 25 ng/mL BMP2 treatment substantially decreased ICAM1 mRNA (Figure 3C) and protein (Figure 3D) levels at 24 h, and 48 h.

### BMP2 upregulates the expression of ID3 in HESCs and primary HDSCs

**FIGURE 4:**
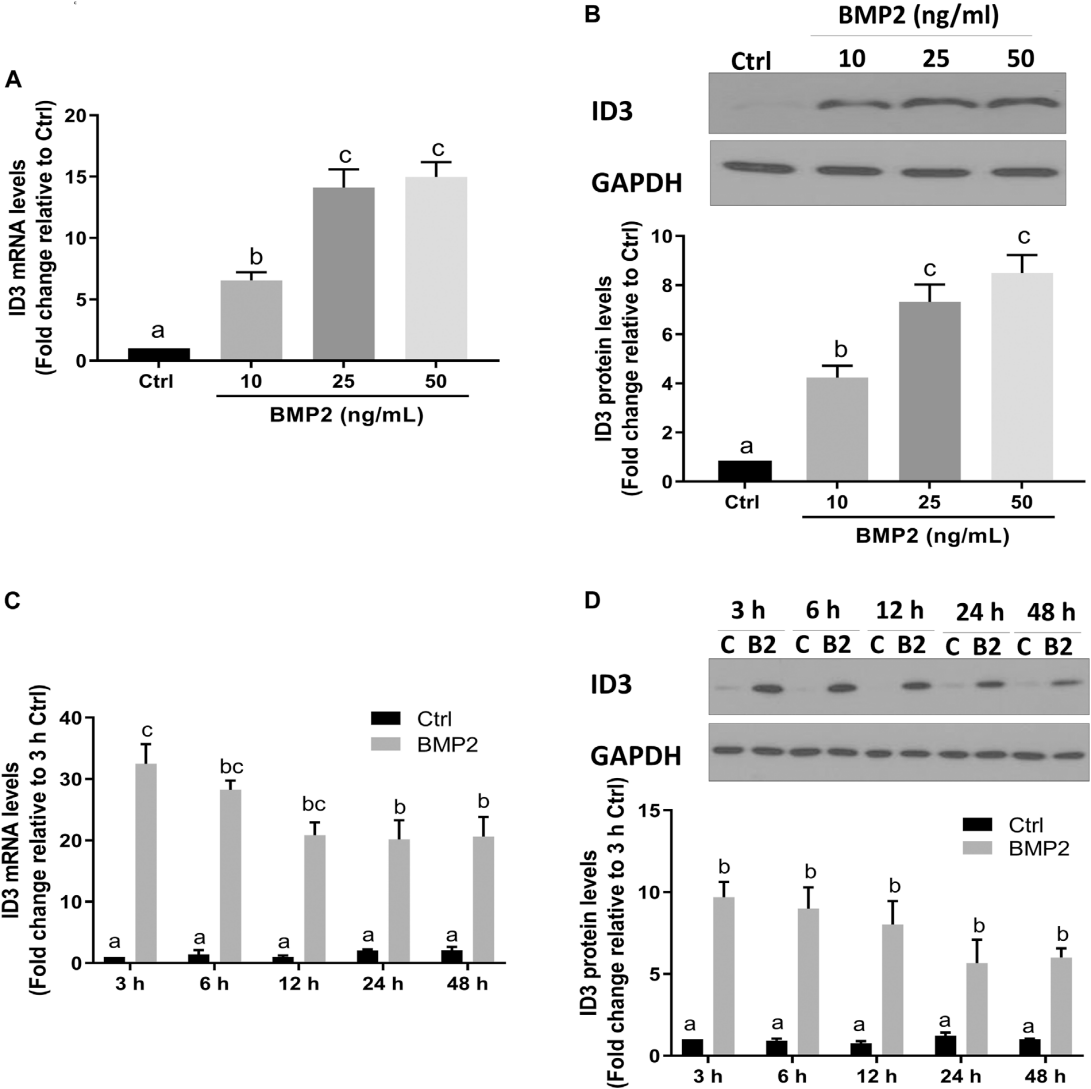
BMP2 upregulates the expression of ID3 in HESCs. (A and B) HESCs were treated with different concentrations (0, 10, 25, 50 ng/mL) of BMP2 for 24 h, and the mRNA **(A)** and protein **(B)** levels of ID3 were examined using RT-qPCR and Western blot analysis, respectively. **(C,D)** HESCs were treated with 25 ng/mL of BMP2 for 3, 6, 12, 24 or 48 h, and the mRNA **(C)** and protein **(D)** levels of ID3 were examined using RT-qPCR and Western blot analysis, respectively. The results are expressed as the mean ± S.E.M. of at least three independent experiments. Different letters indicate a significant difference (*p* < 0.05).

**FIGURE 5:**
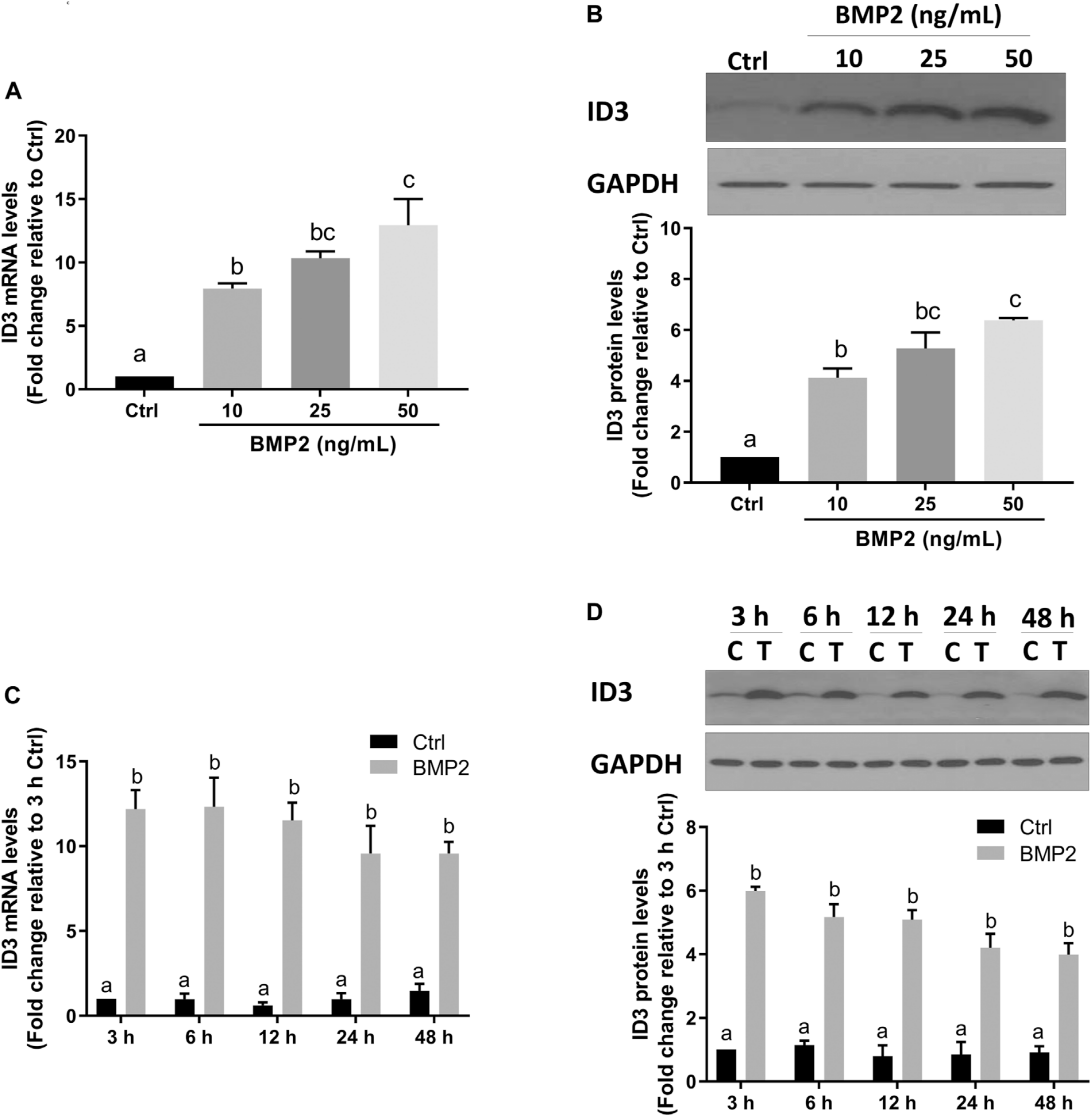
BMP2 upregulates the expression of ID3 by BMP2 in HDSCs. (A and B) HDSCs were treated with different concentrations (0, 10, 25, 50 ng/mL) of recombinant human BMP2 for 24 h, and the mRNA **(A)** and protein **(B)** levels of ID3 were examined using RT-qPCR and Western blot analysis, respectively. **(C,D)** HDSCs were treated with 25 ng/mL of BMP2 for 3, 6, 12, 24 or 48 h, and the mRNA **(C)** and protein **(D)** levels of ID3 were examined using RT-qPCR and Western blot analysis, respectively. The results are expressed as the mean ± S.E.M. of at least three independent experiments. Different letters indicate a significant difference (*p* < 0.05).

Transcriptional regulator inhibitor of DNA-binding/differentiation-3 (ID3) is a member of the helix-loop-helix (HLH) protein family. Several previous studies have demonstrated that ID3 is a downstream target of BMPs [28–30]. To determine if ID3 is a downstream target of BMP2 in this assay system, the expression levels of ID3 in HESCs and HDSCs were assessed using RT‒qPCR and Western blot analysis following BMP2 treatment. As shown in Figure 4, the mRNA (Figure 4A) and protein (Figure 4B) the expression levels of ID3 were remarkably increased after treatment with gradually increased concentrations of BMP2 in HESCs. In time-response experiments, the mRNA (Figure 4C) and protein (Figure 4D) levels of ID3 markedly rose 3 h after 25 ng/ml BMP2 treatment and persisted for 48 h in HESCs. Similar results were noted in the primary HDSCs. Increasing BMP2 doses resulted in a considerable rise in mRNA (Figure 5A) and protein (Figure 5B) expression levels of ID3, and 25 ng/ml BMP2 treatment markedly enhanced the mRNA (Figure 5C) and protein (Figure 5D) levels of ID3 at 3 h and persisted for 48 h in HDSCs. When cells were treated with BMP2 at a concentration of 10 ng/ml, the expression levels of ID3 and ICAM1 were significantly changed both in HESCs and HDSCs, but the decreased range of ICAM1 expression in protein was mild (0.84-fold change relative to Ctrl in HESCs and 0.85-fold change relative to Ctrl in HDSCs). Therefore, we chose a concentration of 25 ng/ml for the follow-up studies to ensure the stability and reproducibility of the experiment.

### ID3 mediates the BMP2-induced downregulation of ICAM1 in HESCs and primary HDSCs

**FIGURE 6:**
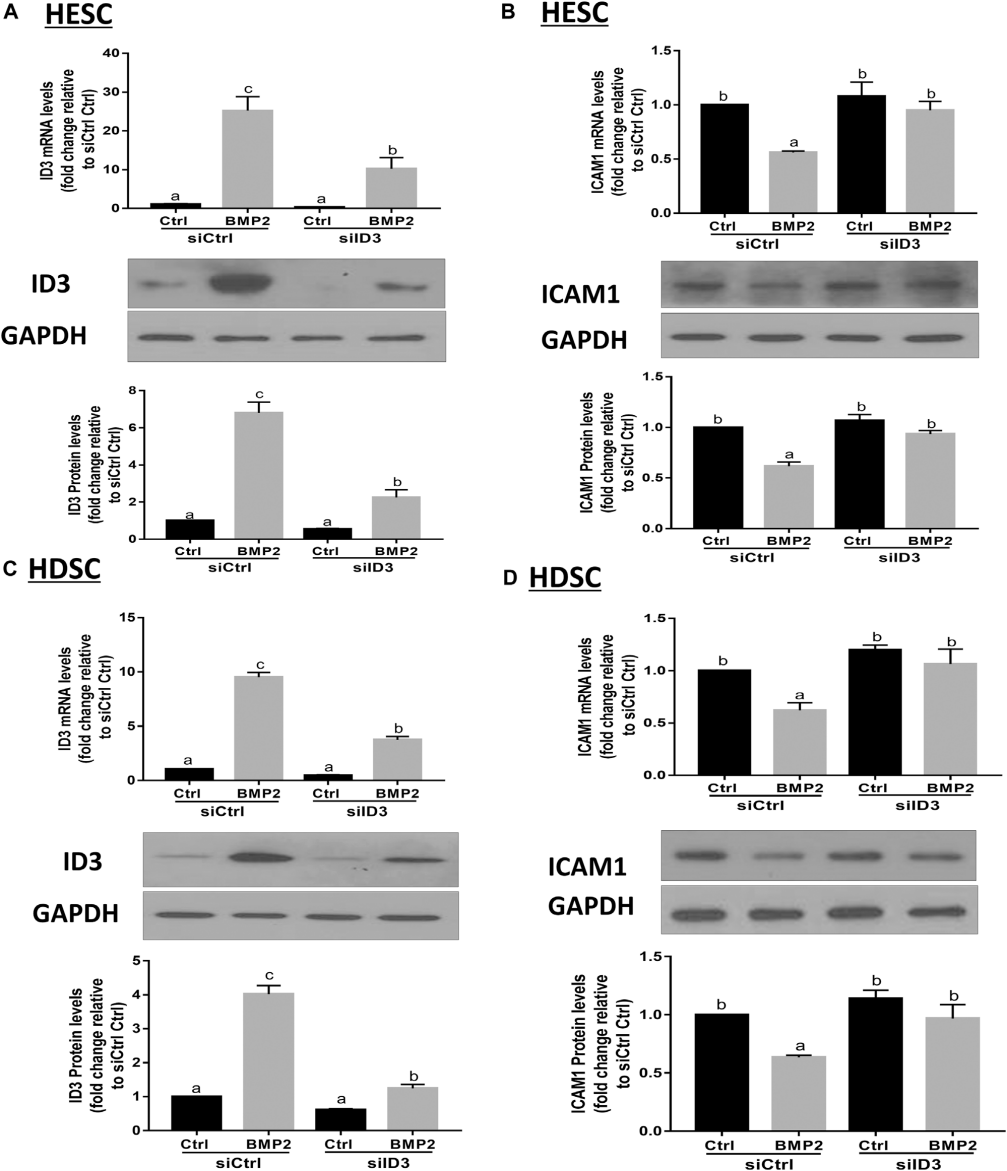
ID3 mediates the BMP2-induced downregulation of ICAM1 expression in HESCs and HDSCs. **(A,B)** HESCs were transfected with 25 nM control siRNA (siCtrl) or 25 nM siRNA targeting ID3 (siID3) for 48 h, and the cells were then treated with vehicle control (Ctrl) or 25 ng/mL BMP2 for an additional 24 h. The mRNA and protein levels of ID3 **(A)** and ICAM1 **(B)** were examined using RT-qPCR and Western blot analysis respectively. **(C,D)** HDSCs were transfected with 25 nM siCtrl or 25 nM siID3 for 48 h, and the cells were then treated with Ctrl or 25 ng/ml of BMP2 for an additional 24 h. The mRNA and protein levels of ID3 **(C)** and ICAM1 **(D)** were examined using RT-qPCRand Western blot analysis, respectively. The results are expressed as the mean ± S.E.M. of at least three independent experiments. Different letters indicate significant a difference (*p* < 0.05).

By transfecting HESCs and primary HDSCs with ID3-targeting siRNA (siID3), siRNA-based knockdown studies were carried out to investigate the functions of ID3 in BMP2-induced downregulation of ICAM1. The HESCs and primary HDSCs were transfected with siID3 for 24 h, starved in serum-free media for 18 h, and then cocultured with recombinant BMP2 (25 ng/mL) for 24 h. Our results showed that knockdown of ID3 significantly reduced the ID3 expression induced by BMP2 at the mRNA and protein levels (Figure 6A) as well as reversed the mRNA and protein levels of the BMP2-induced downregulation of ICAM1 (Figure 6B) in HESCs. Similar findings were also obtained in primary HDSCs (Figures 6C,D). However, silencing ID3 does not seem to alter basal levels of ICAM1. The same phenomenon has been found in some other studies (Li et al., 2019; Li et al., 2021). We believe that the possible reason is that in the non-stimulated HESCs and HDSCs, the expression of ID3 was very low, and silencing ID3 cannot alter the constitutive expression of ICAM1 but can only affect the inducible ICAM1 expression. These findings imply that ID3 mediates the BMP2-induced downregulation of ICAM1 in HESCs and primary HDSCs.

### DMH-1 or dorsomorphin abolishes the BMP2-induced upregulation of ID3 and downregulation of ICAM1 expression in HESCs and primary HDSCs

**FIGURE 7:**
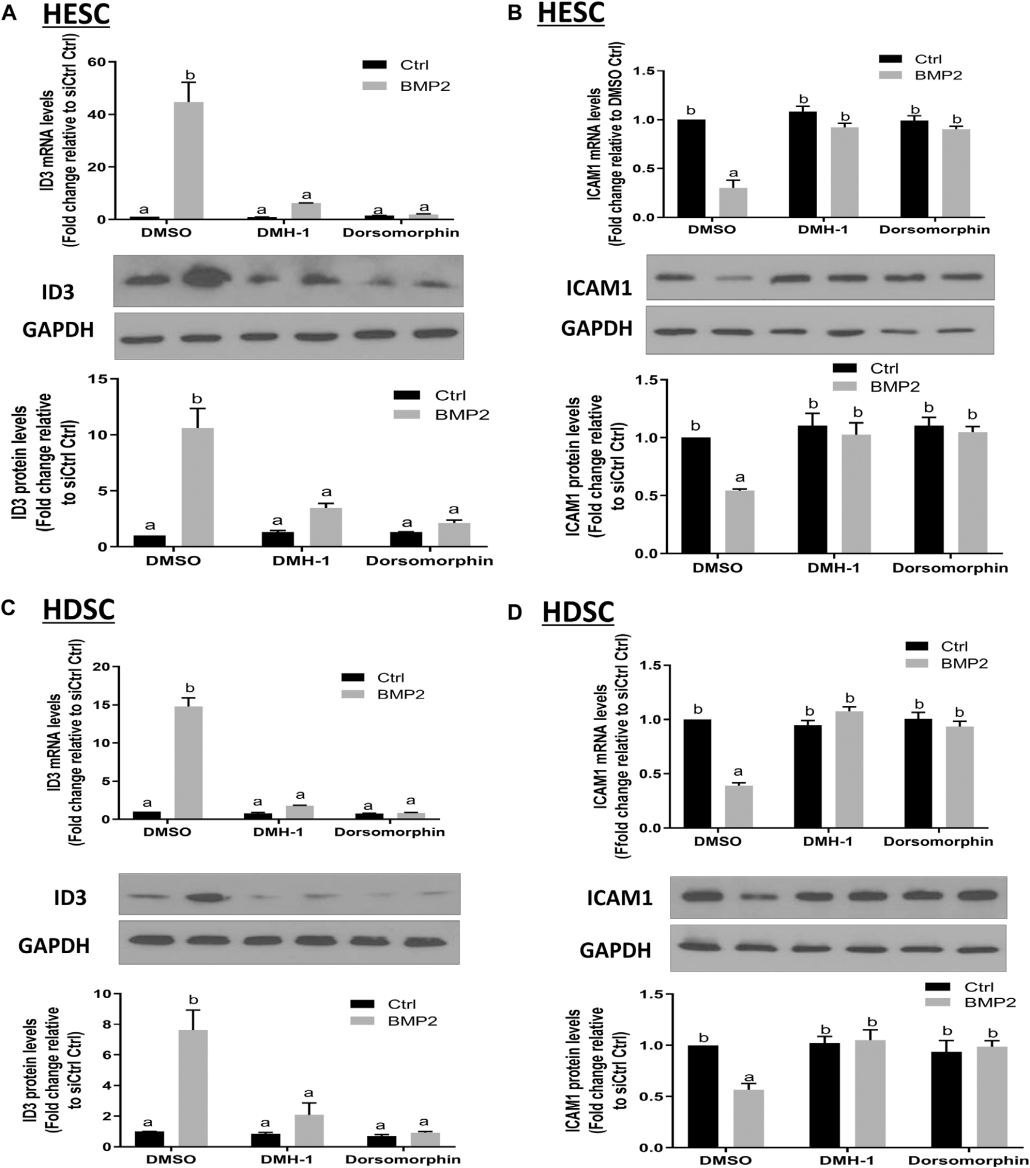
The effects of specific TGF-β type I inhibitors on BMP2-induced upregulation of ID3 expression and downregulation of ICAM1 expression in HESCs and HDSCs. (A and B) HESCs were pretreated with dimethyl sulfoxide (DMSO), DMH-1 (0.25 µM), or dorsomorphin (10 µM) for 1 h, and the cells were then treated with 25 ng/ml BMP2 for an additional 24 h. The mRNA and protein levels of ID3 **(A)** and ICAM1 **(B)** were examined using RT-qPCR and Western blot analysis, respectively. **(C,D)** HDSCs were pretreated with DMSO, DMH-1 (0.25 µM), or dorsomorphin (10 µM) for 1 h, and the cells were then treated with 25 ng/mL BMP2 for an additional 24 h. The mRNA and protein levels of ID3 **(C)** and ICAM1 **(D)** were examined using RT-qPCR and Western blot analysis, respectively. The results are expressed as the mean ± S.E.M. of at least three independent experiments. Different letters indicate a significant difference (*p* < 0.05).

BMPs transduce signals through transmembrane serine/kinases composed of type I (ALK2, ALK3, and ALK6) and type II receptors (Salazar et al., 2016). To further clarify the role of these type I receptors in the BMP2-induced upregulation of ID3 and downregulation of ICAM1 expression in HESCs and primary HDSCs, these two kinds of cells were pretreated with different TGF-β type I receptor inhibitors, namely, DMH-1 (an inhibitor of ALK2 and ALK3) or dorsomorphin (an inhibitor of ALK2, ALK3, and ALK6) for 1 h, followed by treatment with 25 ng/mL BMP2 for another 24 h. The cells in the control group were treated with equal volumes of DMSO, which has been widely used to formulate compounds for cell administration. Several studies have confirmed that there is no interference with the cell experiment if the final concentration of DMSO is controlled within 0.1% (v/v) (Qi et al., 2008; Dludla et al., 2018). In the present study, the final concentration of DMSO is 0.05% (v/v), which is considered to be a safe vehicle of reagents for almost all cells. The results showed that either DMH-1 or dorsomorphin completely abolished BMP2-induced upregulation of ID3 (Figures 7A, C) and downregulation of ICAM1 (Figures 7B, D) mRNA and protein expression in the HESCs (Figures 7A, B) and primary HDSCs (Figures 7C, D). These results indicate that BMP2 regulates the expression of ID3 and ICAM1 through ALK2 and/or ALK3 receptors.

### ALK3 type I receptor mediates the BMP2-induced upregulation of ID3 and downregulation of ICAM1 expression in HESCs and primary HDSCs

**FIGURE 8:**
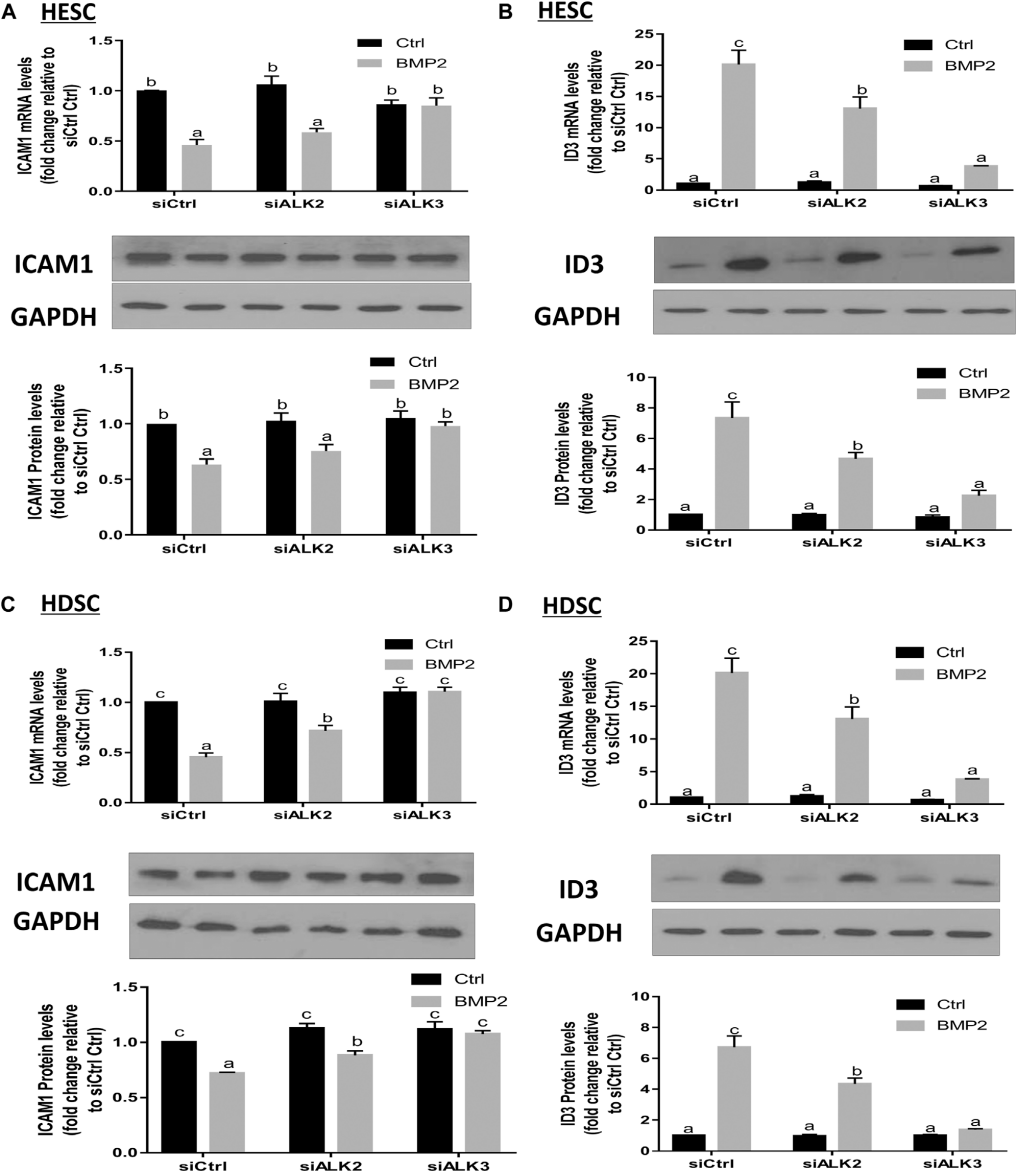
ALK3 type I mediates the BMP2-induced downregulation of ICAM1 and ID3 in HESCs and HDSCs. **(A,B)** HESCs were transfected with 25 nM siCtrl, siRNA targeting ALK2 (siALK2) or siRNA targeting ALK3 (siALK3) for 48 h, and the cells were then treated with Ctrl or 25 ng/mL of BMP2 for an additional 24 h. The mRNA levels of ICAM1 **(A)** and ID3 **(B)** were examined using RT-qPCR and the protein levels of ICAM1 and ID3 were examined using Western blot analysis. **(C,D)** HDSCs were transfected with 25 nM siCtrl, 25 nM siALK2 or 25 nM siALK3 for 48 h, and the cells were then treated with Ctrl or 25 ng/mL BMP2 for an additional 24 h. The mRNA levels of ICAM1 **(C)** and ID3 **(D)** were examined using RT-qPCR, and the protein levels of ICAM1 and ID3 were examined using Western blot analysis. The results are expressed as the mean ± S.E.M. of at least three independent experiments. Different letters indicate significant a difference (*p* < 0.05).

To learn more about the particular type I receptor that BMP2 uses to upregulate ID3 expression and to reduce ICAM1 expression in HESCs and primary HDSCs, the effects of ALK2 or ALK3 were suppressed using a siRNA-based suppression strategy. The cells were pretreated with specific siRNAs targeting ALK2 (siALK2) or ALK3 (siALK3) for 48 h, followed by treatment with 25 ng/mL BMP2 for an additional 24 h. Our results showed that pretreated with siALK2 or siALK3 significantly reduced the target gene expression in HESCs and HDSCs without affecting the expression of the other one, and the expression levels of ALK2 and ALK3 were not affected by BMP2 treatment (Supplementary Figure S2). As shown in Figure 8, knockdown of ALK3 completely abolished the downregulation of ICAM1 (Figure 8A) and upregulation of ID3 (Figure 8B) induced by BMP2 at both the mRNA and protein levels in HESCs. Interestingly, knockdown of ALK2 partially inhibited the BMP2-induced downregulation of ICAM1 (Figure 8C) and upregulation of ID3 expression (Figure 8D) in HDSCs. However, it seemed to have no significant effect on the downregulation of ICAM1 mRNA or protein expression (Figure 8A) induced by BMP2 in HESCs. These results suggest that ALK3 is the major receptor for upregulating ID3 and downregulating ICAM1 expression by BMP2 in HESCs and primary HDSCs.

### SMAD4 mediates the BMP2-induced upregulation of ID3 and downregulation of ICAM1 expression in HESCs and primary HDSCs

**FIGURE 9:**
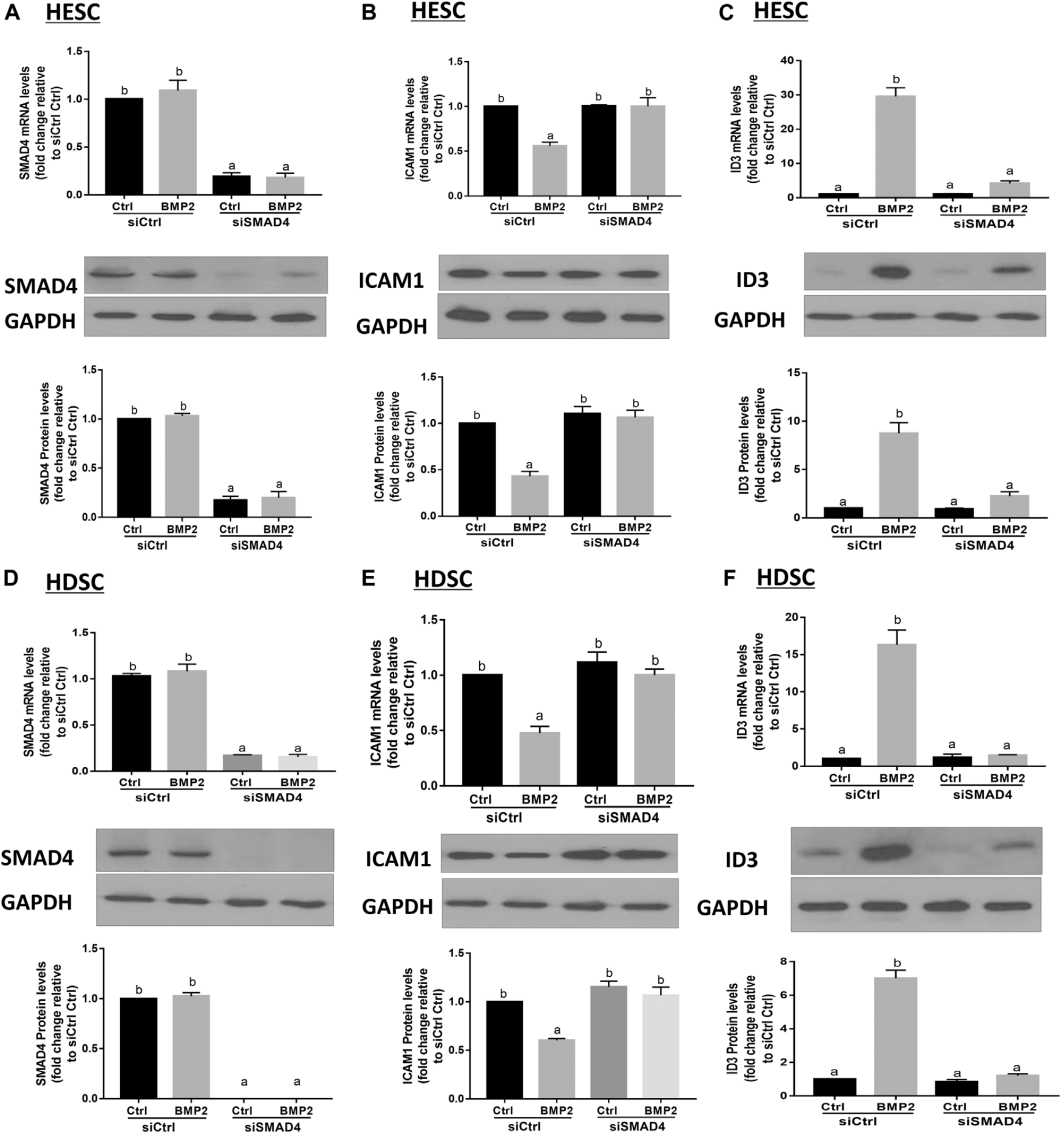
SMAD4 is involved in the BMP2-induced upregulation of ID3 expression and downregulation of ICAM1 expression in HESCs and HDSCs. **(A–C)** HESCs were transfected with 25 nMsiCtrl or 25 nM siRNA targeting SMAD4 (siSMAD4) for 48 h, and the cells were then treated with BMP2 (25 ng/mL) for an additional 24 h. The mRNA and protein levels of SAMD4 **(A)**, ICAM1 (B) and ID3 **(C)** were examined using RT-qPCR and Western blot analysis, respectively. **(D–F)** HDSCs were transfected with 25 nM siCtrl or 25 nM siSMAD4 for 48 h, and the cells were then treated with BMP2 (25 ng/mL) for an additional 24 h. The mRNA and protein levels of SAMD4 **(D)**, ICAM1 **(E)** and ID3 **(F)** were examined using RT-qPCR and Western blot analysis. Respectively. The results are expressed as the mean ± S.E.M. of at least three independent experiments. Different letters indicate significant a difference (*p* < 0.05).

Prior to moving into the nucleus to control target genes, phosphorylated R-SMADs join forces with SMAD4 to create a heterotrimeric transcription factor complex. We transfected HESCs and primary HDSCs with siSMAD4 to inhibit endogenous SMAD4 expression in order to better understand if the SMAD signaling pathway is involved in the BMP2-induced overexpression of ID3 and downregulation of ICAM1. Following a 48-h siSMAD4 transfection, the cells were given 25 ng/mL BMP2 treatment for an additional 24-h period. SMAD4 knockdown effectiveness was confirmed by Western blot and RT-qPCR analysis. Our results showed that the expression of SMAD4 almost eliminated after siSMAD4 transfection and treatment with 25 ng/mL BMP2 did not alter SMAD4 expression in HESCs and HDSCs (Figures 9A, D). The siRNA-mediated depletion of SMAD4 completely abolished the downregulation of ICAM1(Figure 9B) and upregulation of ID3 (Figure 9C) mRNA and protein expression in HESCs. Notably, SMAD4 knockdown also completely abolished BMP2-induced cell activity in HDSCs (Figures 9E, F).

## Discussion

Due to the unclear underlying pathogenesis and the lack of effective interventions, uRPL remains one of the most challenging and frustrating fertility-related diseases. In the present study, we found that mid-secretory endometrium samples obtained from uRPL patients had significantly lower BMP2 and higher ICAM1 levels than fertile controls. Additionally, we demonstrate for the first time that BMP2 suppresses ICAM1 expression through a mechanism reliant on ID3 overexpression both in non-decidual and decidual stromal cells. This information will aid in the development of new pharmacological strategies for unexplained RPL.

There are a large number of cell adhesion molecules in the human endometrium that appear to be necessary to successfully establish the physical interaction between the embryo and the endometrium (van Mourik et al., 2009). One of the best-characterized cell adhesion molecules is ICAM1, which is a ligand for integrin molecule 1 (LFA-1). The expression of ICAM1 has previously been examined in endometrial stromal cells and endometrial epithelial cells in multiple species (Blois et al., 2005; Defrere et al., 2005; Lecce et al., 2011). A previous study demonstrated that ICAM1 was substantially increased in the uterine epithelium and the stroma of high-stress sensing induced abort-prone mice compared to control mice, resulting in more LFA-1-expressing lymphocytes being recruited from the blood into the uterus. Neutralization of the adhesion molecules ICAM1/LFA-1 radically eliminated the effect of stress on the embryonic abortion (Blois et al., 2005). Further research revealed that upregulated ICAM1 in the decidua promoted Th1 polarization *via* mature dendritic cells, leading to Th1/Th2 imbalance (Blois et al., 2005), which is known to contribute to the pathogenesis of RPL. Notably, evidence from a clinical study showed that elevated ICAM1 levels detected by ELISA in human endometrial tissue correlated with idiopathic RPL (Comba et al., 2015). Similar results were obtained by examining the RNA and protein expression levels of ICAM1 in endometrial tissue obtained from uRPL patients and normal fertile women in our study. At the same time, we also detected that BMP2 expression in the endometrium of these two groups exhibited an opposite phenotype to ICMA1. It has been demonstrated that the upregulation of ICAM1 in HESCs can be induced by various inflammatory mediators such as IL-1 β (Vigano et al., 1994), TNF-α (Thomson et al., 1999), and INF-γ (Mangioni et al., 2005). However, the regulation of ICAM1 by BMP2 has not yet been reported. Definitive evidence shows that BMP2 is essential for pregnancy establishment and maintenance by regulating blastocyst implantation, uterine decidualization and placental/fetal development (Lee et al., 2007; Yi et al., 2021). Transcriptome analysis revealed that BMP2 targets primarily play a key role in regulating cell adhesion and extracellular matrix (ECM) transformation in human endometrium (Yi et al., 2021). By controlling the expression of IGFBP3, our prior work also showed that BMP2 contributes to endometrial remodeling in human non-decidual and decidual stromal cells (Luo et al., 2020). In this study, our *in vitro* analysis showed that the expression of ICAM1 in HESCs and primary HDSCs can also be regulated by BMP2 *via* ID3.

The inhibitors of DNA binding proteins (ID) are dominant negative antagonists of basic helixloop-helix (bHLH) transcription factors. To date, four ID family proteins have been identified in mammalian cells and have been demonstrated to be expressed in the uterine endometrium as well as the maternal-fetal interface (Han et al., 2018). Our studies showed that ID1 (Luo et al., 2020), ID2 (Supplementary Figure S3), and ID3 (Figure 4; Figure 5) are among the most significantly upregulated genes upon stimulation of BMP2. Further studies based on the siRNA-based knockdown experiments found that it was ID3 but not ID1 or ID2 that mediated BMP2-induced downregulation of ICAM1 in HESCs and primary HDSCs (Figure 6 and Supplementary Figure S4). ID3 expression can be differentially regulated by members of the TGF-β superfamily in various cell types; for example, TGF-β1 represses ID3 expression in adult neural stem/precursor cells (Bohrer et al., 2015), while TGF-β1 increases ID3 mRNA and nuclear ID3 protein levels in immortalized human granulosa cells (Li et al., 2019). In addition, other TGF-β superfamily members, such as BMP4 and BMP6, upregulate ID3 expression in a range of different cell lines, including embryonic stem cells, human B progenitor cells, intestinal stem cells, and neuronal stem cells (Hollnagel et al., 1999; Kersten et al., 2006; Hu et al., 2021). Importantly, a previous study revealed that ID3 was dramatically upregulated by BMP2 in adult neural stem/precursor cells and was essential for BMP2- induced differentiation of neural stem/precursor cells into astrocytes (Bohrer et al., 2015). In this study, our findings add to growing evidence that the overexpression of ID3 induced by BMP2 was required for BMP2-suppressed ICAM1 expression in HESCs and primary HDSCs. Furthermore, we explored the underlying molecular mechanism by which BMP2 regulates the ID3 and ICAM1 expression by pretreating HESCs and primary HDSCs with different TGF-β type I receptor inhibitors, including DMH-1 (an inhibitor of ALK2/3) and dorsomorphin (an inhibitor of ALK2/3/6) prior to BMP2 treatment. Our results show that the inhibitors DMH-1 and dorsomorphin can significantly eliminate the upregulation of ID3 and downregulation of ICAM1 induced by BMP2, but the siRNA-mediated gene downregulation provided more accurate evidence that it is ALK3 rather than ALK2 mainly responsible for the downstream pathway of BMP2 induction. We suppose that although ALK3 and ALK2 are paralogous genes and both are downstream receptors of BMPs, their protein primary structures are about 10% different, which may lead to some functional divergence. This will be interesting research content in the future. Ligand‒receptor complexes induce downstream signaling in a SMAD-dependent manner following BMP2 binding to specific receptors (Shi and Massague, 2003). SMAD1/5 is thought to be the main downstream signaling pathway that mediates BMP2 signaling pathway in HESCs and primary HDSCs (Zhang et al., 2020; Zhang et al., 2022). Upon phosphorylation of type I receptors in the majority of tissues, phosphorylated SMAD1/5 bind to a common SMAD (SMAD4) to create a heterotrimer complex, which translocates into the nucleus to control the expression of target genes. Here, knocking down SMAD4 totally reversed the effects of BMP2 on ID3 and ICAM1 expression, suggesting that SMAD4 is required for BMP2-induced intracellular signaling in HESCs and primary HDSCs.

**FIGURE 10:**
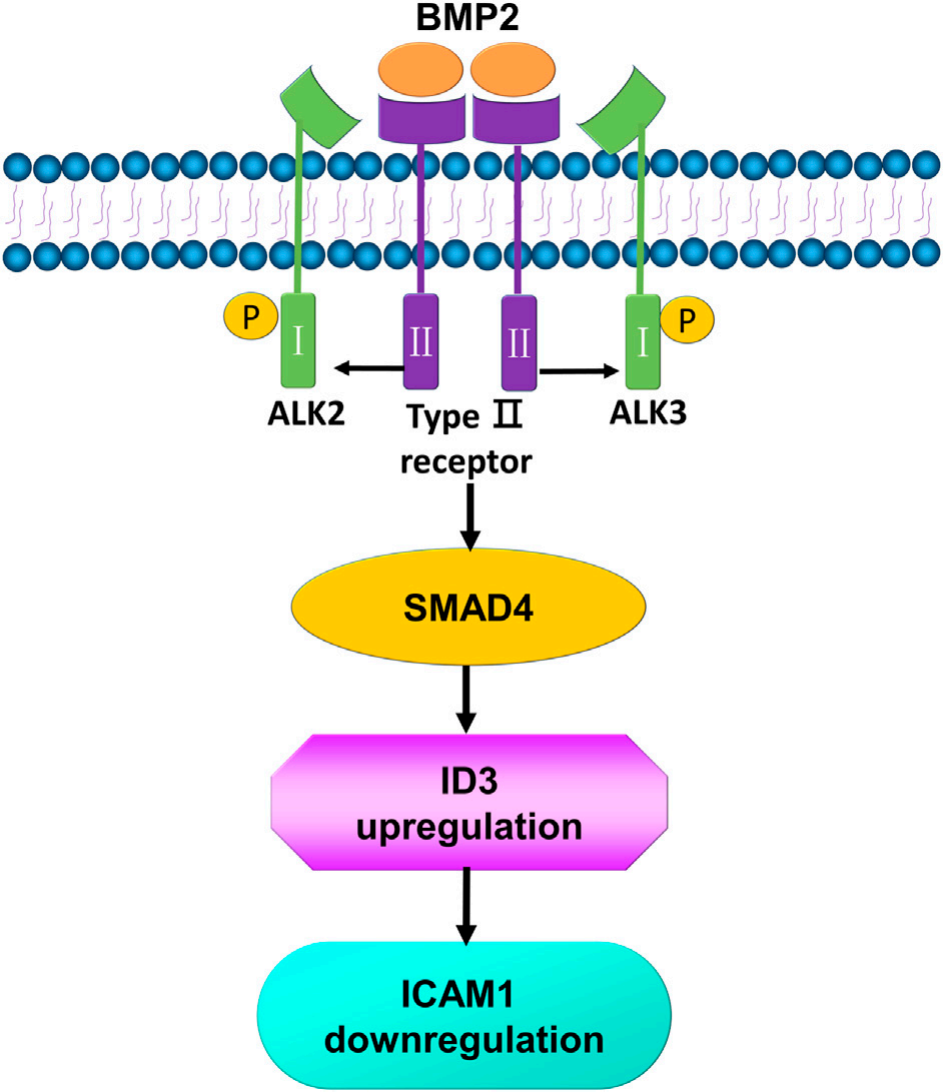
Schematic diagram of the proposed molecular mechanisms by which BMP2 downregulates the expression of ICAM1 in human endometrial stromal cells and decidual cells. BMP2 binds to a pair of ALK3 type 1 receptor and BMP type II receptors, leading to the activation of canonical R-SMADs, which are associated with a common SMAD (SMAD4) and further increases the transcription of ID3. The increase in ID3 suppresses the expression of ICAM1.

In conclusion, our data reveal that downregulation of BMP2 in the endometrium may contribute to the pathogenesis of uRPL by increasing ICAM1 expression *via* the ALK3-SMAD4-ID3 signaling pathway (Figure 10). These findings not only deepen the understanding of the molecular regulatory mechanisms of ICAM1 expression in the human endometrium but also suggest that it may be possible to improve the pregnancy outcomes in patients with uRPL by regulating the local expression of BMP2 or ICAM1 in the endometrium.

## Data Availability

The original contributions presented in the study are included in the article/Supplementary Material, further inquiries can be directed to the corresponding authors.
