# Supplementary material for: ID3 mediates BMP2-induced downregulation of ICAM1 expression in human endometiral stromal cells and decidual cells
Source: Front Cell Dev Biol. 2023 Feb 24;11:1090593. doi: 10.3389/fcell.2023.1090593 (PMC9998904; doi:10.3389/fcell.2023.1090593)
Supplement: Supplementary file 1 [file Table1.DOCX]

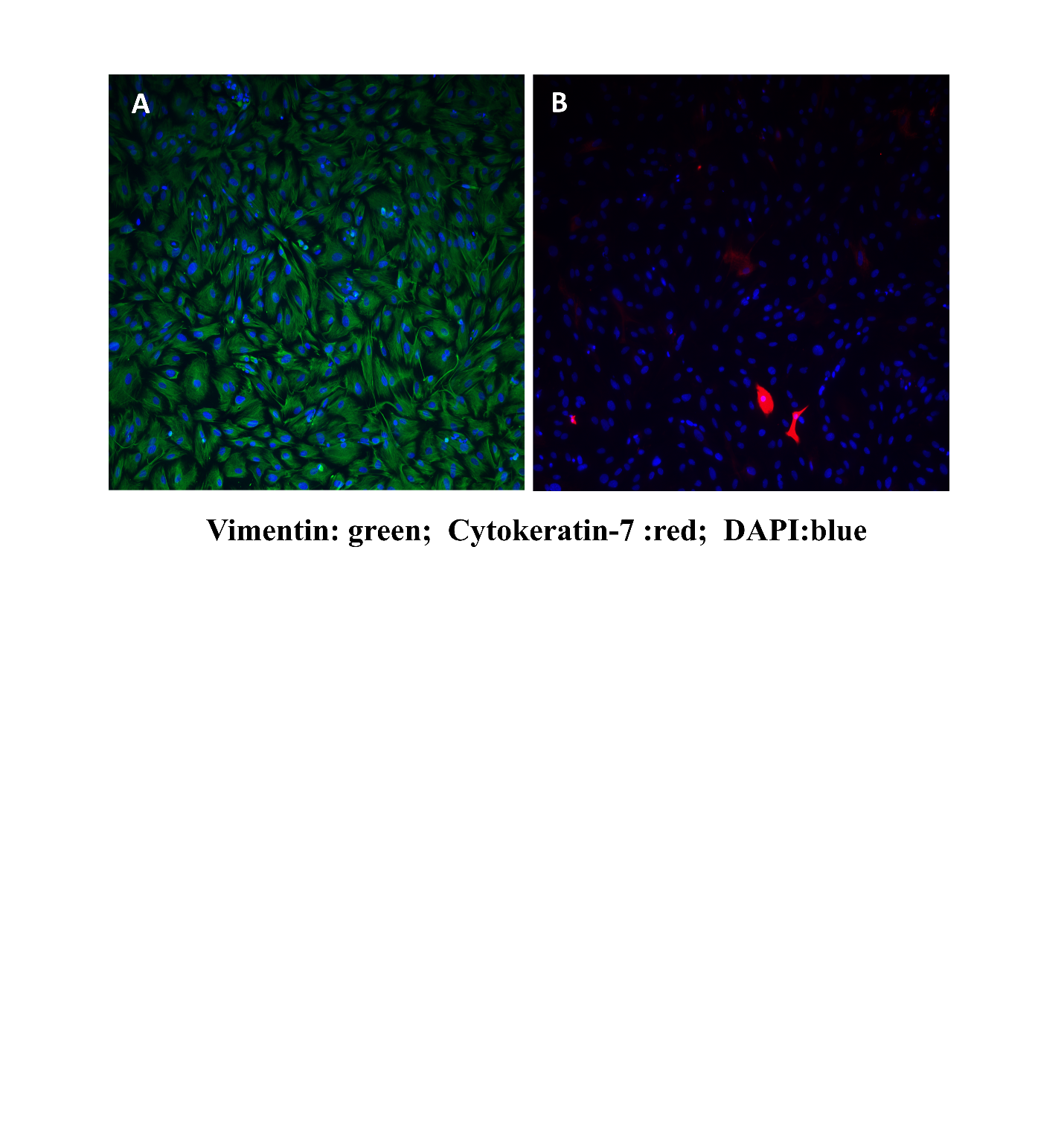


Supplementary Fig.1. Immunofluorescent staining of vimentin (Mesenchymal cells-specific, signal is shown in green, A), and cytokeratin-7 (epithelial cells-specific, the signal is shown in red, B) in Primary HDSCs isolated from first-trimester human decidual tissues. Nuclear DNA was labeled in blue with 4’,6-Diamidino-2’-phenylindole (DAPI). Primary HDSCs were seeded at 18 ×18 mm2 glass coverslips in 6 well culture dishes for 24 h and then the cells were fixed in 4% paraformaldehyde for 30 min at room temperature. After being incubated in 3% H2O2 in phosphate-buffered saline (PBS) for 30 min, the cells were blocked with serum-free protein block (Dako) for 60 min at room temperature. Coverslips were incubated with a primary antibody against mouse cytokeratin 7(MAB3554, Millipore, Temecula, ON, Canada.) and vimentin (3390S, NEB, Whitby, ON, Canada) for 60 min, and then incubated with a fluorochrome-conjugated secondary antibody Alexa Fluor 488 or 594 goat anti-mouse IgG, respectively (1:200 dilution, Vimentin: green, cytokeratin: red, Life Technology,  HYPERLINK "http://en.wikipedia.org/wiki/Carlsbad,_California" \o "Carlsbad, California" \t "_blank" Carlsbad, CA, USA) for 60 min at room temperature. Finally, after DAPI staining, the cells were observed and photographed under an inverted microscope (Olympus BX61, Breinigsville, PA, USA). Magnification: 200×
