# Supplementary material for: ID3 mediates BMP2-induced downregulation of ICAM1 expression in human endometiral stromal cells and decidual cells
Source: Front Cell Dev Biol. 2023 Feb 24;11:1090593. doi: 10.3389/fcell.2023.1090593 (PMC9998904; doi:10.3389/fcell.2023.1090593)
Supplement: Supplementary file 2 [file Table2.DOCX]

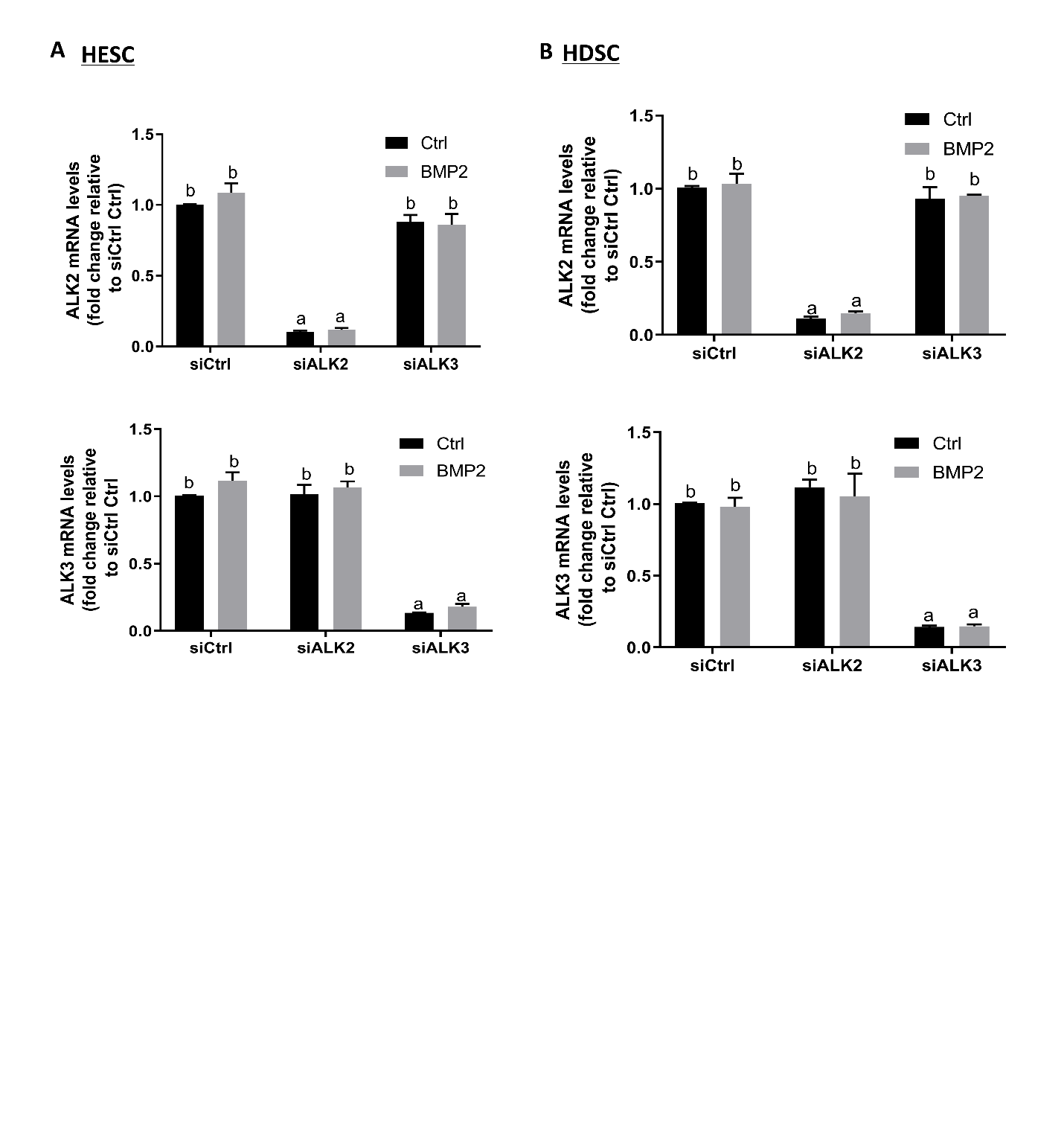


Supplementary Fig. 2. The knockdown efficiency of ALK2 and ALK3 examined by TaqMan RT**‒**qPCR. (A and B) HESCs (A) and HDSCs (B) were transfected with 25 nM siCtrl, siRNA targeting ALK2 (siALK2) or siRNA targeting ALK3 (siALK3) for 48 h, and the cells were then treated with Ctrl or 25 ng/mL of BMP2 for an additional 24 h. The expression levels of ALK2 and ALK3 were examined by TaqMan RT**-**qPCR. The results are expressed as the mean ± S.E.M. of at least three independent experiments. Different letters indicate significant a difference (*P* < 0.05).
