# Supplementary material for: ID3 mediates BMP2-induced downregulation of ICAM1 expression in human endometiral stromal cells and decidual cells
Source: Front Cell Dev Biol. 2023 Feb 24;11:1090593. doi: 10.3389/fcell.2023.1090593 (PMC9998904; doi:10.3389/fcell.2023.1090593)
Supplement: Supplementary file 3 [file Table3.DOCX]

Supplementary Figure 3. Dose responses of ID2 and ID4 mRNA expressions in HESCs and primary HDSCs treated with BMP2. (A and B) HESCs (A) and HDSCs (B) were treated with different concentrations (10, 25, or 50 ng/ml) of recombinant human BMP2 for 24 h. The mRNA levels of ID2 and ID4 were examined using RT-qPCR. The results are expressed as mean ± S.E.M. of at least three independent experiments. Different letters indicate a significant difference (P < 0.05).
