# Supplementary material for: ID3 mediates BMP2-induced downregulation of ICAM1 expression in human endometiral stromal cells and decidual cells
Source: Front Cell Dev Biol. 2023 Feb 24;11:1090593. doi: 10.3389/fcell.2023.1090593 (PMC9998904; doi:10.3389/fcell.2023.1090593)
Supplement: Supplementary file 4 [file Table4.DOCX]

Supplementary Figure 4. The effect of siID1 and siID2 on the downregulation of ICAM1 induced by BMP2 in HESCs and primary HDSCs. HESCs (A and C) or HDSCs (B and D) were transfected with 25 nM siCtrl, siID1 or siID2 for 48 h and then treated with vehicle (Ctrl) or 25 ng/ml BMP2 for another 24 h. The mRNA levels of ICAM1, ID1 and ID2 were examined using RT-qPCR. The results are expressed as mean ± S.E.M. of at least three independent experiments. Different letters indicate a significant difference (P < 0.05).
